# Acute Motor Axonal Neuropathy (AMAN) Variant of Guillain-Barré Syndrome Following Influenza Vaccination as the Initial Presentation of Systemic Lupus Erythematosus With Lupus Nephritis

**DOI:** 10.7759/cureus.111062

**Published:** 2026-06-17

**Authors:** Njoud Alshammari, Mnieaam Z Aldugieman, Mohammed S Alshammar, Hamad Alrasheedi, Ahmed H Alreshidi

**Affiliations:** 1 Rheumatology, King Salman Specialist Hospital, Hail, SAU; 2 Neurology, King Salman Specialist Hospital, Hail, SAU; 3 Internal Medicine, King Salman Specialist Hospital, Hail, SAU; 4 Internal Medicine, King Khalid Hospital, Hail, SAU

**Keywords:** acute flaccid paralysis, aman, guillain-barré syndrome, influenza vaccination, lupus nephritis, systemic lupus erythematosus

## Abstract

Guillain-Barré syndrome (GBS) is an acute immune-mediated polyradiculoneuropathy that commonly follows infections and, less frequently, vaccination. Although peripheral nervous system involvement may occur in systemic lupus erythematosus (SLE), GBS as the initial manifestation of SLE is uncommon, particularly in adolescents. Herein, we describe the case of a previously healthy 17-year-old Saudi female patient who presented with rapidly progressive bilateral lower-limb weakness, areflexia, bilateral foot drop, and urinary retention occurring approximately two weeks after an upper respiratory tract infection and one week after receiving an influenza vaccine. Nerve conduction studies and electromyography demonstrated a demyelinating-axonal polyneuropathy compatible with the acute motor axonal neuropathy (AMAN) variant of GBS. During hospitalization, she was also found to have inflammatory ascites, pleural effusions, Coombs-positive anemia, proteinuria, positive antinuclear antibodies, markedly elevated anti-double-stranded DNA (anti-dsDNA) antibodies, and hypocomplementemia. Renal biopsy confirmed minimal mesangial lupus nephritis (ISN/RPS Class I). Magnetic resonance imaging of the brain and entire spine was unremarkable. The patient received intravenous immunoglobulin, corticosteroids, hydroxychloroquine, and supportive treatment. Although cyclophosphamide was considered because of concerns regarding neuropsychiatric lupus, it was ultimately withheld. The patient achieved complete neurological recovery without cyclophosphamide. This case highlights the occurrence of AMAN variant GBS shortly after influenza vaccination as the initial presentation leading to the diagnosis of SLE and lupus nephritis.

## Introduction

Guillain-Barré syndrome (GBS) is an acute immune-mediated polyradiculoneuropathy characterized by rapidly progressive weakness, diminished or absent reflexes, and variable sensory and autonomic dysfunction [1]. The disorder most commonly develops following respiratory or gastrointestinal infections, with approximately two-thirds of patients reporting a preceding infectious illness. However, vaccination may act as a potential trigger in some cases [2]. Several electrophysiological subtypes have been recognized, including acute inflammatory demyelinating polyneuropathy (AIDP), acute motor axonal neuropathy (AMAN), and acute motor-sensory axonal neuropathy (AMSAN). AMAN is characterized by selective motor axonal involvement and is often associated with antecedent immune stimulation [1]. Systemic lupus erythematosus (SLE) is a chronic multisystem autoimmune disease with heterogeneous clinical manifestations. Neurological involvement is well recognized and may affect both the central and peripheral nervous systems [3]. However, GBS remains a rare manifestation of SLE and may occur either as part of neuropsychiatric lupus or as a coincidental autoimmune neuropathy in a patient with lupus. In some instances, GBS may precede the diagnosis of SLE and represent the first clinical manifestation of the disease [4,5]. The coexistence of GBS and newly diagnosed SLE poses a significant diagnostic challenge. Distinguishing classic GBS from lupus-associated peripheral neuropathy is essential because management approaches differ considerably. While GBS is primarily treated with intravenous immunoglobulin or plasma exchange, severe lupus-related neuropathies often require aggressive immunosuppression with high-dose corticosteroids and cyclophosphamide [4].

Herein, we report the case of a previously healthy adolescent female who developed the AMAN variant of GBS shortly after influenza vaccination and an upper respiratory tract infection. Her neurological presentation ultimately led to the diagnosis of SLE with lupus serositis and biopsy-proven Class I lupus nephritis.

## Case presentation

A 17-year-old Saudi female patient with no significant past medical history presented to the emergency department with rapidly progressive bilateral lower-limb weakness and inability to walk for two days. Two weeks prior to presentation, she developed symptoms of an upper respiratory tract infection and self-medicated with over-the-counter medications. During the same period, she also received a seasonal influenza vaccine at school. Approximately one week later, she developed dysuria and acute urinary retention, prompting evaluation at a peripheral hospital, where imaging reportedly suggested a ureteric stone. A Foley catheter was inserted for bladder decompression.

On physical examination, she appeared ill but was conscious, alert, and fully oriented. Her heart rate was 116 beats/minute, while the remaining vital signs were stable. Neurological examination demonstrated complete paralysis of both lower limbs (MRC grade 0/5), generalized hypotonia, bilateral foot drop, and absent deep tendon reflexes. Sensory examination was normal. Upper-limb motor and sensory functions were intact. Persistent urinary retention required continued Foley catheterization. Abdominal examination revealed generalized tenderness and marked distension without signs of peritoneal irritation.

Initial laboratory investigations demonstrated marked leukocytosis, with a peak white blood cell count of 44.16 × 10⁹/L, anemia, elevated inflammatory markers, a ferritin level of 736 ng/mL, an elevated lactate dehydrogenase (LDH) level of 468 U/L, and a strongly positive direct Coombs test (+3). Renal and liver function tests were within normal limits. Urinalysis revealed 2+ proteinuria, and 24-hour urinary protein excretion was 1.2 g/day (Table 1).

**Table 1:** Hematological, biochemical, and urinary investigations CRP: C-reactive protein, ESR: erythrocyte sedimentation rate, LDH: lactate dehydrogenase.

Investigation	Result	Reference range/interpretation
White blood cell count	Peak 44.16 × 10⁹/L	4.0-11.0 × 10⁹/L
Hemoglobin	8.6 g/dL	12.0-16.0 g/dL
Platelet count	198-315 × 10⁹/L	150-450 × 10⁹/L
ESR	47 mm/hour	<20 mm/hour
CRP	18.8 mg/L	<5 mg/L
Ferritin	736 ng/mL	Female: 13-150 ng/mL
Serum LDH	468 U/L	135-225 U/L
Direct Coombs test	Positive (+3)	Negative
Urinalysis	+2 protein	Negative
24-hour urine protein	1.2 g/day	<150 mg/day

Lumbar puncture revealed clear, colorless cerebrospinal fluid with a white blood cell count of 4 cells/µL, a red blood cell count of 0 cells/µL, a glucose concentration of 2.8 mmol/L, a protein concentration below 20 mg/L, and an LDH level of 32 U/L. Gram stain and culture were negative, while cytological examination demonstrated only a few lymphocytes without evidence of malignancy. Because of marked abdominal distension and ascites, diagnostic paracentesis was performed. The ascitic fluid was turbid and yellow, with a white blood cell count of 5.11 × 10⁶/L consisting predominantly of neutrophils (57%). The serum-ascites albumin gradient was 0.8 g/dL. Fluid analysis demonstrated elevated protein (48 g/L), albumin (24 g/L), LDH (>750 U/L), and amylase (>1500 U/L) levels, consistent with inflammatory exudative ascites. Cytological examination revealed reactive mesothelial cells mixed with abundant neutrophils and no malignant cells. Fluid cultures were negative (Table 2).

**Table 2:** Cerebrospinal fluid and ascitic fluid analysis CSF: cerebrospinal fluid, LDH: lactate dehydrogenase, RBC: red blood cell, SAAG: serum-ascites albumin gradient, WBC: white blood cell.

Investigation	Result	Reference range/interpretation
Cerebrospinal fluid analysis		
CSF appearance	Clear, colorless	Normal
CSF WBC	4 cells/µL	0-5 cells/µL
CSF RBC	0 cells/µL	0-5 cells/µL
CSF glucose	2.8 mmol/L	2.2-3.9 mmol/L
CSF protein	<20 mg/L	150-450 mg/L
CSF LDH	32 U/L	<40 U/L
CSF Gram stain	Negative	Negative
CSF culture	Negative	No growth
CSF cytology	Few lymphocytes; no malignancy	No malignant cells
Ascitic fluid analysis		
Appearance	Turbid, yellow	Normally clear, straw-colored
WBC count	5.11 × 10⁶/L	Elevated; inflammatory process
Neutrophils	57%	Elevated inflammatory predominance
Lymphocytes	23%	Variable
Monocytes	20%	Variable
RBC count	0 × 10⁶/L	Absent
Culture	Negative	No growth
Cytology	Reactive mesothelial cells with abundant neutrophils; no malignancy	No malignant cells
SAAG	0.8 g/dL	<1.1 g/dL suggests exudative ascites
Protein	48 g/L	>25 g/L consistent with exudative ascites
Albumin	24 g/L	Used for SAAG calculation
Glucose	3.2 mmol/L	Typically approximates serum glucose
LDH	>750 U/L	Elevated; inflammatory/exudative process
Amylase	>1500 U/L	Usually <100 U/L

Autoimmune investigations revealed a positive antinuclear antibody (ANA) titer of 1:80, markedly elevated anti-double-stranded DNA (anti-dsDNA) antibodies (109 IU/mL), low C3 (0.75 g/L), and low C4 (0.06 g/L). Additional autoantibodies, including ANCA, anti-cyclic citrullinated peptide (anti-CCP) antibodies, anticardiolipin antibodies, beta-2 glycoprotein antibodies, anti-SSA, anti-SSB, anti-Sm, anti-Scl-70, and anti-Jo-1 antibodies, were negative. Serum protein electrophoresis demonstrated polyclonal hypergammaglobulinemia. Magnetic resonance imaging (MRI) of the brain and entire spinal cord with contrast was unremarkable (Figure 1).

**Figure 1:**
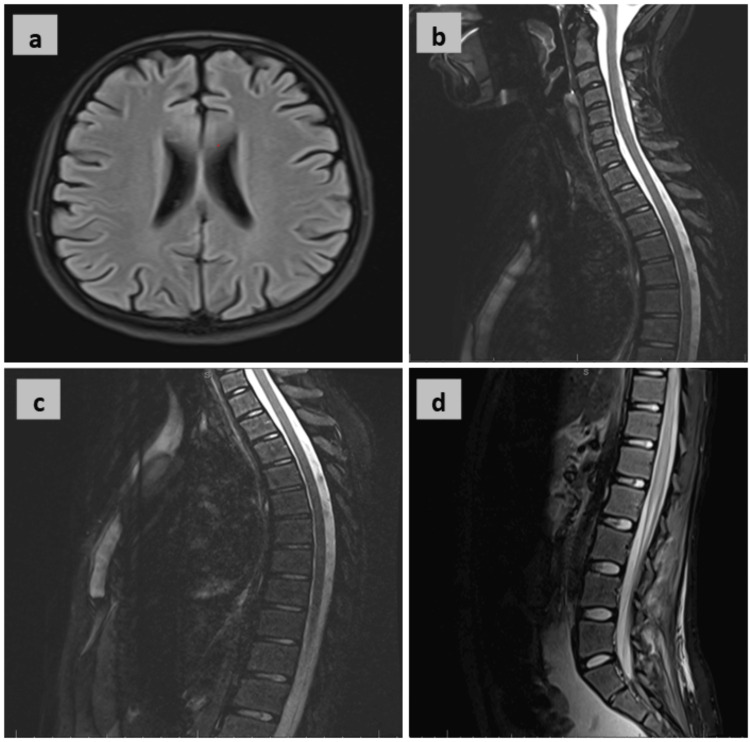
Magnetic resonance imaging (MRI) of the brain and the entire spine with contrast (a) Axial fluid-attenuated inversion recovery (FLAIR) MRI of the brain demonstrating normal size ventricles with preserved cortical sulci and no abnormal signal intensity. (b) Sagittal T2-weighted MRI of the cervical spine showing normal alignment and preserved vertebral body height, with no evidence of spinal cord compression, atrophy or focal lesion. (c) Sagittal T2-weighted MRI of the thoracic spine demonstrating normal spinal cord morphology and signal intensity with intact intervertebral disc and no evidence of disc herniation, canal stenosis, or intramedullary pathology. (d) Sagittal T2-weighted MRI of the lumbar spine demonstrating normal spinal cord morphology and signal intensity with intact intervertebral disc and no evidence of disc herniation.

Electrophysiological studies demonstrated absent F-waves, absent sural sensory responses, low-amplitude deep peroneal responses, delayed tibial distal motor latency, reduced motor conduction velocities, and denervation potentials in the lower limbs. These findings were interpreted as being consistent with the AMAN variant of GBS. Renal biopsy demonstrated normal glomerular architecture on light microscopy without sclerosis, crescents, or interstitial fibrosis. However, immunofluorescence revealed full-house mesangial immune complex deposition (IgG, IgA, C3, and C1q), consistent with minimal mesangial lupus nephritis (ISN/RPS Class I) (Figure 2 and Figure 3).

**Figure 2:**
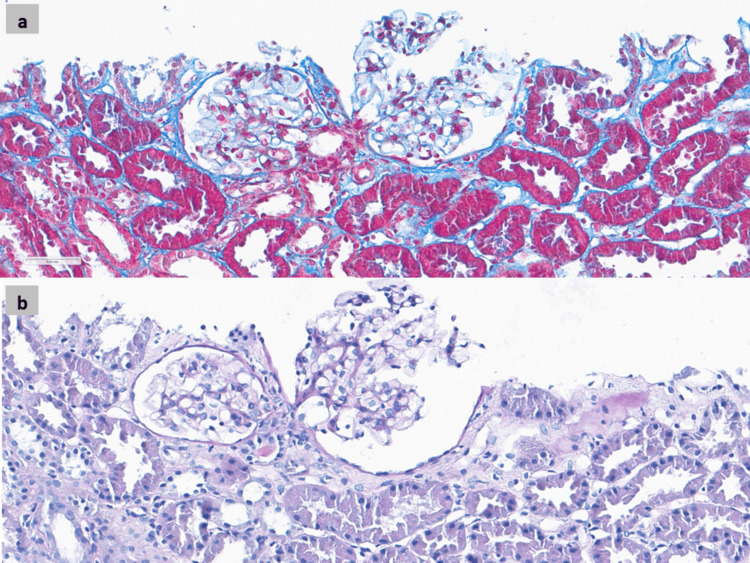
(a) Renal biopsy stained with H&E stain, demonstrating normal renal parenchyma with no significant glomerular abnormalities on light microscopy. (b) Renal biopsy stained with PAS stain, demonstrating normal renal parenchyma with no significant glomerular abnormalities on light microscopy

**Figure 3:**
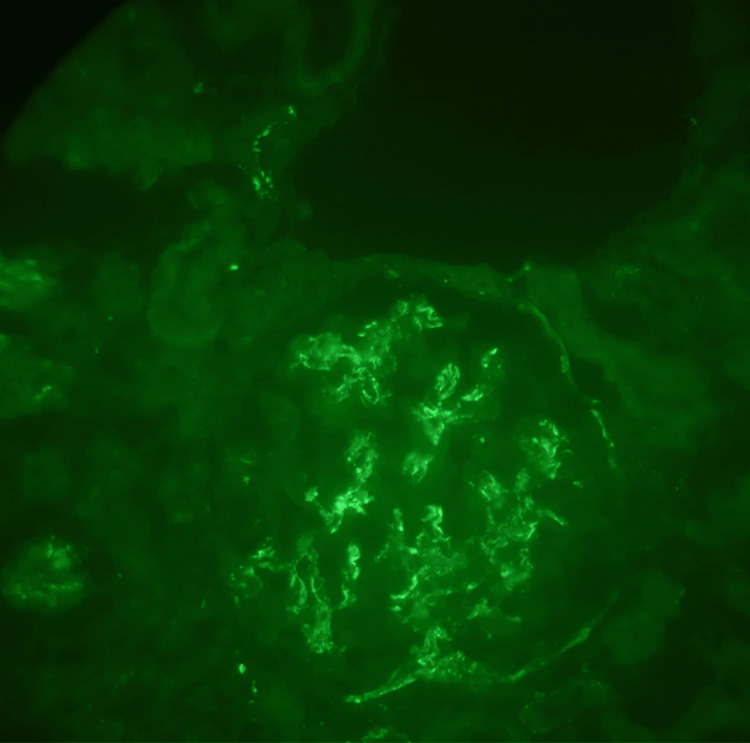
Renal biopsy immunofluorescence showing positive glomerular mesangial staining for IgG (2+), IgA (1+), C3 (2+), C1q (2+), and Kappa light chains (2+)

The patient was initially managed as having GBS with intravenous immunoglobulin and pulse methylprednisolone. Broad-spectrum antibiotics were administered because of concern for peritonitis. Following confirmation of SLE, hydroxychloroquine and corticosteroid therapy were continued. Cyclophosphamide was considered because of concern for lupus-related neuropathy but was deferred due to the development of a urinary tract infection and vaginal candidiasis. During follow-up, the patient demonstrated progressive neurological recovery and ultimately regained full motor strength, independent ambulation, and normal bladder function without receiving cyclophosphamide.

## Discussion

Only a limited number of cases of GBS as the initial presentation of SLE have been reported in the literature. A literature review by Bhoi et al. identified only 28 published cases of GBS presenting as the initial manifestation of SLE [4]. The present case illustrates an uncommon and diagnostically challenging presentation of newly diagnosed SLE complicated by acute flaccid paralysis. The principal challenge was distinguishing between peripheral nervous system involvement secondary to lupus and GBS occurring concomitantly with SLE. Neurological manifestations occur in 15% to 95% of patients with SLE, although peripheral neuropathy is relatively uncommon [3]. Acute inflammatory demyelinating polyneuropathy and axonal variants resembling GBS have been reported as initial manifestations of lupus, particularly in young females. Several mechanisms have been proposed, including immune complex deposition, vasculitic nerve injury, antibody-mediated neuronal damage, and shared autoimmune pathways [4].

Our patient met the 2019 EULAR/ACR classification criteria for SLE, with a positive ANA, elevated anti-dsDNA antibody levels, hypocomplementemia, Coombs positivity, serositis, and biopsy-proven lupus nephritis [6]. Simultaneously, she exhibited several features highly suggestive of GBS, including antecedent respiratory infection, rapidly progressive symmetrical weakness, areflexia, autonomic dysfunction manifested by urinary retention, and supportive electrophysiological findings [7]. The AMAN variant is characterized by selective involvement of motor axons and often follows infectious triggers. Although anti-ganglioside antibody testing was unavailable, the electrophysiological findings of low-amplitude motor responses, absent F-waves, and denervation potentials strongly supported this diagnosis [8]. Interestingly, cerebrospinal fluid analysis did not demonstrate albuminocytologic dissociation. However, normal CSF findings are well recognized during the early stages of GBS and do not exclude the diagnosis [9].

An important diagnostic consideration was lupus-associated peripheral neuropathy. Acute axonal neuropathy related to SLE is most often attributed to vasculitic nerve injury and typically presents with prominent neuropathic pain, asymmetric weakness, sensory deficits, and a multifocal mononeuritis multiplex pattern. In contrast, our patient had painless, rapidly progressive, symmetrical lower-limb paralysis, generalized areflexia, preserved sensory examination, urinary retention, and electrophysiological evidence of diffuse motor axonal involvement without a multifocal pattern. These features favored classic AMAN-type GBS occurring in the setting of newly diagnosed SLE rather than primary lupus vasculitic neuropathy. In the present case, both an antecedent upper respiratory tract infection and influenza vaccination occurred within the recognized risk window preceding symptom onset. Therefore, causality cannot be attributed to either exposure. Respiratory infections remain the most common precipitating factor for GBS, whereas the attributable risk following influenza vaccination is exceedingly low. It is possible that either exposure or a combination of both contributed to the immune activation preceding the development of AMAN. An unusual feature of this case was the markedly low cerebrospinal fluid protein concentration (<20 mg/L). Although the absence of albuminocytologic dissociation can occur in early GBS, particularly within the first week of symptom onset, CSF protein concentrations are generally expected to remain within the normal range rather than being markedly reduced.

Another notable feature was the presence of inflammatory ascites and pleural effusions. Lupus peritonitis is an uncommon manifestation of serositis and may occasionally be the presenting feature of SLE [10]. The low SAAG, elevated protein concentration, neutrophil-predominant inflammatory fluid, and negative microbiological studies were most consistent with lupus-associated serositis rather than portal hypertension or infectious peritonitis. Similarly, the presence of Class I lupus nephritis confirmed renal involvement despite preserved renal function. The optimal treatment of GBS associated with SLE remains controversial. Previous reports have described successful treatment with IVIG, plasmapheresis, corticosteroids, cyclophosphamide, rituximab, or combinations thereof [4]. For instance, Laarhoven et al. reported complete resolution in a patient with GBS and lupus nephritis following treatment with cyclophosphamide [11]. Another report described a young male patient with a GBS-like neuropathy and ascites who initially responded dramatically to IVIG therapy. However, the patient experienced a recurrence eight months later and did not respond to IVIG after relapse [12].

In our patient, cyclophosphamide was strongly considered because of concern for neuropsychiatric lupus. However, complete neurological recovery occurred without cyclophosphamide, suggesting that the neuropathy behaved more like classic post-infectious GBS than severe lupus-mediated peripheral neuropathy. This observation highlights the importance of individualized treatment decisions and careful longitudinal assessment. The temporal relationship between respiratory infection, influenza vaccination, and symptom onset deserves mention. Both antecedent infection and vaccination have been associated with GBS, although the absolute risk following influenza vaccination remains extremely low.

## Conclusions

This case highlights an unusual presentation of SLE characterized by the AMAN variant of GBS, lupus serositis with inflammatory ascites and pleural effusions, and Class I lupus nephritis. Distinguishing GBS from lupus-related peripheral neuropathy can be challenging because of overlapping clinical and immunological features. In this patient, the presence of an antecedent infection, characteristic electrophysiological findings, autonomic dysfunction, and complete recovery without cyclophosphamide favored a diagnosis of concomitant GBS rather than severe neuropsychiatric lupus. Recognition of this rare overlap is important because management strategies and prognostic implications differ substantially. A multidisciplinary approach involving neurology, rheumatology, nephrology, and rehabilitation teams is essential to achieve optimal outcomes in such complex cases.
